# Valorization of Moroccan *Pistacia lentiscus* L. Leaves: Phytochemical and *In Vitro* Antioxidant Activity Evaluation Compared to Different Altitudes

**DOI:** 10.1155/2022/6367663

**Published:** 2022-03-26

**Authors:** Abderrahmane Hadini, Amal Azdimousa, Amine Khoulati, Khalid El bekkaye, Ennoumane Saalaoui

**Affiliations:** ^1^Laboratory of the Improvement of Agricultural Production, Biotechnology and the Environment, Faculty of Sciences, Mohammed First University, BP 717, Oujda 60000, Morocco; ^2^Laboratory of Bioresources, Biotechnology, Ethnopharmacology and Health, Faculty of Sciences, Mohammed First University, BP 717, Oujda 60000, Morocco

## Abstract

This study examined the secondary metabolite content and the antioxidant activities of hydromethanolic *P. lentiscus* L. leaves extracts at different altitudes. The results indicated that the contents of polyphenols and flavonoids were significantly (*p* ≤ 0.05) high in the low altitude, unlike the Chl (chlorophyll), tannins, and ascorbic acid, which were reported to have higher content in the high altitude. These results showed that the *P. lentiscus* L. is more adaptable to higher elevations than low elevation, where the plant was probably stressed. On the other hand, the analyses of correlation between the antioxidant activity and phytochemical content affirmed that the antiradical activity (DPPH) correlated with the content of polyphenols; however, the total antioxidant activity is correlated with the flavonoid content. These results revealed the importance of *P. lentiscus* L. leaves as a natural antioxidant and gave an idea of the altitude effect on the biochemical parameters of leaves.

## 1. Introduction

Morocco is considered one of the richest countries in flora and fauna biodiversity in the Mediterranean basin. Its geographical position and orographic and climatic factors provides favorable ecological conditions for developing rich and varied flora. Aromatic and medicinal plants constitute an important part of this biological diversity. They are part of the population's natural resources directly improving their income and still include an essential source for traditional healers [1]. The northeast of Morocco is also considered an important reservoir of floristic diversity, especially aromatic and medicinal plants [2]. Their biological effects approved in recent years, *in vivo* and *in vitro*, and bioactive substances discovered in modern medicine are assimilated from ethnopharmacological surveys [3]. The reasons that support the use of medicinal plants are their low cost, availability, and moderate toxic effects compared to conventional therapy [4]. We cite *P. lentiscus* L., also known as mastic tree or Lentisk, among these aromatic and medicinal plants [5]. The genus *Pistacia* belongs to the Anacardiaceae family and encompasses about twenty species, including the evergreen leaves or deciduous shrubs and small trees ranging from 5 to 15 m in height [6]. *P. lentiscus* L. is known for its resin biosynthesis [7] and contains anthocyanins [8]. The chemical composition of essential oils mastic gum, leaves, unripe, and ripe fruit contains more than 250 compounds [9] characterized by a large fraction of monoterpene hydrocarbons during flowering. Some studies have highlighted the potential benefit against inflammation and infections of *P. lentiscus* L. [10–12]. In addition, the high content of polyphenols present in the extracts makes them attractive against chronic and degenerative diseases and as nutraceuticals in human health [11]. The essential oil and gum of the plant have been widely used as flavoring additives for beverages and foods and in traditional medicines [13]. Pistachio species are commonly used in food industries, such as adherent production, alcohols, non-alcoholic refreshments, cosmetics, dentistry as a filling ingredient, and toothpaste production [14]. *P. lentiscus* L. leaf extracts have been widely studied for their antimicrobial and antimutagenic activities [15, 16] as well as for their antioxidant capacity [17]. In addition, mastic oil exerted antiproliferative and proapoptotic effects against human leukemia and inhibited vascular endothelial growth factors [13].

However, little research is devoted to studying the effect of the environment on secondary metabolites of *P. lentiscus* L. leaves. This work aims to determine the environmental impact, specifically the altitude, on the leaf's content of certain biochemical indices and the leaf's antioxidant activity of *P. lentiscus* L. distributed in the northeast of Morocco at different altitudes.

## 2. Materials and Methods

### 2.1. Plant Material

*P. lentiscus* L. was determined by the Department of Waters and Forests, Ministry of Agriculture, Maritime Fisheries, Sustainable Development, and Waters and Forests. The leaves were collected, in June 2021, from two zones (Z) with two sites (S) for each zone: the Debdou forest (Z1) 33° 59′ 09″ N, 3° 03′ 04″ W and the Beni Snassen forest (Z2) 34°46′48.45″ N 02°34′48.03″ W. Samples were taken from two individuals chosen randomly within the two zones with two different altitudes for each zone (4 altitudes). The altitudes are as follows: S1.1 = 1400 m, S1.2 = 1120 m for Z1 and S2.1 = 780 m, S2.2 = 720 m For Z2. The samples were cleaned to eliminate damaged, diseased, or infected branches and leaves with pests, and then were dried in an oven at 50°C for 48 h, milled and conserved in polyethylene bags at −20 C until subsequent use.

### 2.2. Hydromethanolic Extract Preparation

Each sample of *P. lentiscus* L. leaves of the four sites was extracted with methanol (80%, v/v). The extract was filtered after maceration for 48 h with an agitator maintained at room temperature. Then, concentrations of 50 mg/mL in methanol (80%, v/v) were prepared and stored at −20°C until each use.

### 2.3. Biochemical Indices

#### 2.3.1. Photosynthetic Pigment

The content of Chl a, Chl b, and total Chl was determined by grinding approximately 0.2 g of the fresh leaf sample in 20 mL of acetone (80%). Then, the mixture was placed for 48 h at room temperature in the dark [18]. The absorbances (Abs) are then read using a spectrophotometer at 663, 645, and 652 nm. The Chl a, b, and total Chl contents were calculated using the following formula:

(1) $$\begin{array}{c} \text{Chl a}\left(\mu \text{g}/\text{mL}\right)=12.21\times {\text{Abs}}_{663}-2.81\times {\text{Abs}}_{645};\\ \text{Chl b}\left(\mu \text{g}/\text{mL}\right)=20.13\times {\text{Abs}}_{645}-5.03\times {\text{Abs}}_{663};\\ \text{Total Chl }=\text{Chl a}+\text{Chl b}. \end{array}$$

#### 2.3.2. Total Polyphenols

The total polyphenol contents of the four sites' leaves were determined by following the Folin–Ciocalteu method described by Velioglu et al. [19], with three repetitions for each site. About 500 *μ*L of the diluted extract from each sample was mixed with 3.75 mL of distilled water and 250 *μ*L of the Folin–Ciocalteu reagent. After 5 min, about 0.5 mL of sodium carbonate solution (20%) was added. Afterward, the mixture was incubated in the dark for 30 min, and the absorbance was then measured at 760 nm using the spectrophotometer. Total polyphenol content was expressed in milligrams of gallic acid equivalent (GAE) by 100 g of dry matter (mg GAE/100 g DM).

#### 2.3.3. Flavonoids

The flavonoid contents were estimated by following the aluminum chloride colorimetric method described by Koolen et al. [20] with three repetitions for each site. About 500 *μ*L of the diluted extract was mixed with 500 *μ*L of the methanolic solution of aluminum chloride (2% p/v). The absorbances are then measured at 430 nm, after incubation at room temperature for 40 min. The flavonoid content was expressed in milligrams of rutin equivalent (RE) by 100 g of dry matter (mg RE/100 g DM).

#### 2.3.4. Condensed Tannins

The content of condensed tannins was determined by the vanillin method described by Julkunen [21], with three replicates for each site. About 50 *μ*L of each extract is added to 1500 *μ*l of the 4% vanillin-methanol solution. Then a volume of 750 *μ*l of concentrated HCl is added. The mixture obtained is left to react at room temperature for 20 minutes. The absorbance was then measured at 550 nm. Condensed tannin concentrations were expressed in micrograms of cyanidin equivalent (CE) by 100 g of dry matter (*μ*g CE/100 g DM).

#### 2.3.5. Ascorbic Acid

The ascorbic acid (AscA) content was estimated using the modified method of Mau et al. [22], with three replicates. About 1 g of the dried leaves are added to 10 mL of oxalic acid (1%) followed by stirring for 15 min. After filtration, 3 mL of the filtrate was mixed with 1 mL of 2,6-dichlorophenolindophenol (DCPIP) 5 mM; the absorbance was then measured at 515 nm after 15 s. The concentrations of AscA are expressed in milligrams of AscA equivalent by 100 g of dry matter (mg AscAE/100 g DM).

### *2.4. In vitro* Antioxidant Activity

The *in vitro* antioxidant activity of *P. lentiscus* L. leaves extracts was assessed using the 1,1diphenyl-2-picrylhydrazyl (DPPH) radical scavenging assay and phosphomolybdate assay (Total antioxidant).

#### 2.4.1. DPPH Radical Scavenging Assay

To determine the scavenging activity of the DPPH-free radical of leaves, a concentration of 1 mg/mL of the first extract concentration (50 mg/mL) is prepared. The antioxidant activity is determined by the modified method described by Braca et al. [23], with three replicates. About 0.1 mL of the extract was added to 2.9 mL of DPPH (6.10^−5^) in methanol, and the Abs were then measured at 517 nm after incubation for 30 min at room temperature. The distilled water was used as a control. The percentage inhibition activity is calculated according to the following equation:

(2) $$\begin{array}{c} \left(\%\right)\text{Inhibition activity}\left(\text{DPPH}\right)=\frac{\left(\left(\text{Abs control}-\text{Abs extract}\right)\right)}{\text{Abs control}}\times 100. \end{array}$$

#### 2.4.2. Phosphomolybdate Assay

The phosphomolybdate assay (total antioxidant activity) was measured by the method suggested by Umamaheswari and Chatterjee [24] with three repetitions. About 100 *μ*L of the extract (1 mg/mL) was added to 1 mL of the phosphomolybdate reagent (0.6 M sulfuric acid, 28 mM sodium phosphate, and 4 mM ammonium molybdate). The solution is incubated in a water bath at 95°C for 90 min, and Abs was measured at 695 nm. The results are expressed as milligram equivalents of *α*-tocopherol by 100 g dry matter (mg ETOC/100 g DM).

### 2.5. Statistical Analysis

All graphs and results are made using SPSS Statistics 17.0 software. Data were expressed as mean ± standard deviation. The Student-Newman–Keuls (SNK) test was used to rank the means using letters with significant differences. ANOVA one way test is used to analyze the variance of the results with altitude as a factor. Pearson correlation determines the relationship between the secondary metabolites analyzed and antioxidant activity. Regression is used to determine the dependence of tannin content and altitude as a factor.

## 3. Results and Discussion

### 3.1. Photosynthetic Pigment

**Figure 1:**
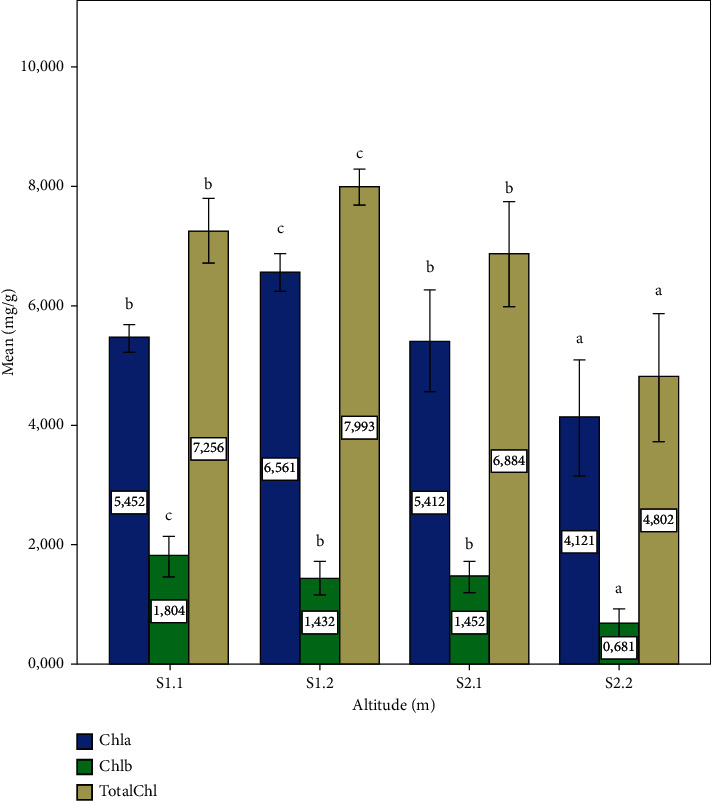
Chlorophyll a, b, and total chlorophyll content the *P. lentiscus* L. leaves at different altitudes. Data are means of three replicates. Vertical bars represent the standard deviation of the mean. Values not sharing a common letter indicate a significant difference at *p* ≤ 0.05, based on the SNK test. Chl a: Chlorophyll a, Chl b: Chlorophyll b, and Total Chl: total chlorophyll. S: site, S1.1 = 1400 m, S1.2 = 1120, S2.1 = 780 m, S2.2 = 720 m.

The highest Chl a, Chl b, and total Chl contents are observed in the samples from site: S1.2 (Altitude: 1120 m), with a significant difference (*p* ≤ 0.05) compared to the chlorophyll contents of the other sites. However, the minimum values are observed in the samples from site 4 (Altitude: 720 m) with a significant difference (*p* ≤ 0.05) (Figure 1). Generally, the highest chlorophyll contents were observed at high altitudes. In nature, chlorophyll molecules are found in several forms [25], the most common being a and b. While chlorophyll is characteristic of plants more exposed to light [26], the concentrations of chlorophyll a and b depend on environmental factors, such as light exposure, temperature, and humidity which vary with altitudes [27]. Chl a and Chl b bind to light-collecting complex (LHC) proteins via weak non-covalent bonds [28]. The light energy captured by the LHC protein is transferred to the thylakoid chloroplast [29]. In our current study, the Chl a content was higher than the Chl b content at the four sample collection sites. In addition, minimum values in total Chl are observed on samples from site 2 of zone 2. The low chlorophyll content can be considered as an adaptive protective mechanism in the event of plant stress [30, 31]. Chlorophyll reduction may also result in LHC protein degradation and disruption and degradation of the envelope of thylakoids and chloroplasts [28, 29]. On the other hand, the high chlorophyll content of Z1 showed that the plants are less exposed to stress conditions than plants in zone Z2. These high Z1 content may be due to the upward activation of enzymes responsible for the regulation and photosynthetic carbon reduction and protection of chloroplasts against oxidative damage.

### 3.2. Total Polyphenols

**Figure 2:**
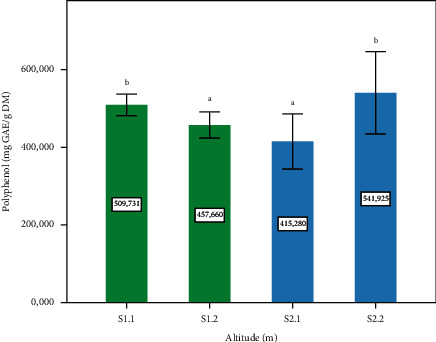
Total polyphenols content of the *P. lentiscus* L. leaves at different altitudes. Data are means of three replicates. Vertical bars represent the standard deviation of the mean. Values not sharing a common letter indicate a significant difference at *p* ≤ 0.05, based on the SNK test. GAE: gallic acid equivalent. DM: dry matter. S: site, S1.1 = 1400 m, S1.2 = 1120, S2.1 = 780 m, S2.2 = 720 m.

The maximum values of polyphenols content are observed in the plants of the two sites S1.1 and S2.2, presenting the altitude of 1400 m and 720 m, respectively, with a significant difference (*p* ≤ 0.05), compared to sites S1.2 and S2.1 (1120 m and 780 m, respectively) (Figure 2). The results obtained in our study are in close agreement with those reported by Rodriguez-Perez et al. [32], who identified different phenolic compounds in *P. lentiscus* L. leaves and are also confirmed by Boucheffa et al. [33]. In comparison with other authors, Gardeli et al. [34] found that the polyphenol content was 588 mg GAE/g in leaves of *P. lentiscus.* Other studies reported by Djeridane et al. [35] (23.5 mg EAG/g DM) and Atmani et al. [36] (136.25 ± 18.9 mg CE/g DM) showed low levels of phenols compared to our result. Other studies have been conducted on Lentisk have demonstrated that the polyphenol content of leaf extracts depends on several factors such as altitude, and the process of the extraction is applied [37, 38]. However, phenolic acids are directly involved in the response of plants to different types of stress. Improved metabolism of phenylpropanoids and the number of phenolic compounds can be affected by various environmental factors: synthesis of isoflavones and several flavonoids generated when plants are infected or damaged, as well as under low-stress conditions [39, 40]; this can be explained by the high content of polyphenols in the leaves at the S2.2.

### 3.3. Flavonoids

**Figure 3:**
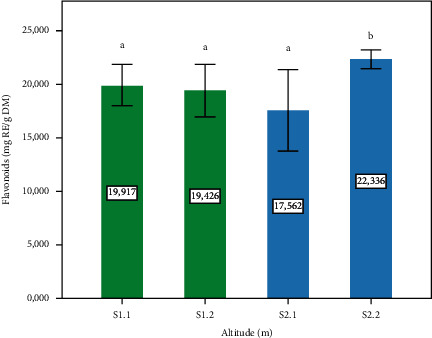
Flavonoid content of the *P. lentiscus* L. leaves at different altitudes. Data are means of three replicates. Vertical bars represent the standard deviation of the mean. Values not sharing a common letter indicate a significant difference at *p* ≤ 0.05, based on the SNK test. RE: rutin equivalent. DM: dry matter. S: site, S1.1 = 1400 m, S1.2 = 1120, S2.1 = 780 m, S2.2 = 720 m.

Figure 3 represents the content of flavonoids observed on the leaves of *P. lentiscus*. The highest range is marked in site S2.2 (Altitude: 720 m) with a significant difference (*p* ≤ 0.05) compared to sites S1.1, S1.2, and S2.1 (1400 m, 1120 m, and 780 m, respectively); flavonoids, belong to a large group of polyphenolic compounds, are part of the polyphenol synthesis pathway and help the plant against oxidative stress situations, which explains the results which are compatible with the variations in polyphenol contents on the 4 sites. In comparison with other studies, flavonoid contents in *P. lentiscus* L. leaves were 12.93 ± 1.69 mg/g and 8.21 ± 0.09 mg/g, which was found by Atmani et al. [36] and Krimat et al. [41], respectively. Indeed, flavonoids rely on many biological activities, including antiviral, antispasmodic, antitumor, antiplatelet aggregation, antiallergic, anti-inflammatory, antihypertensive, and antimicrobial activities [42, 43]. Furthermore, the high content of flavonoids is exploited by representing raw materials for various potential products, including pharmaceuticals and nutraceuticals based on their traditional uses [44]. This also explains its traditional use and the various scientific studies carried out.

### 3.4. Condensed Tannins

**Figure 4:**
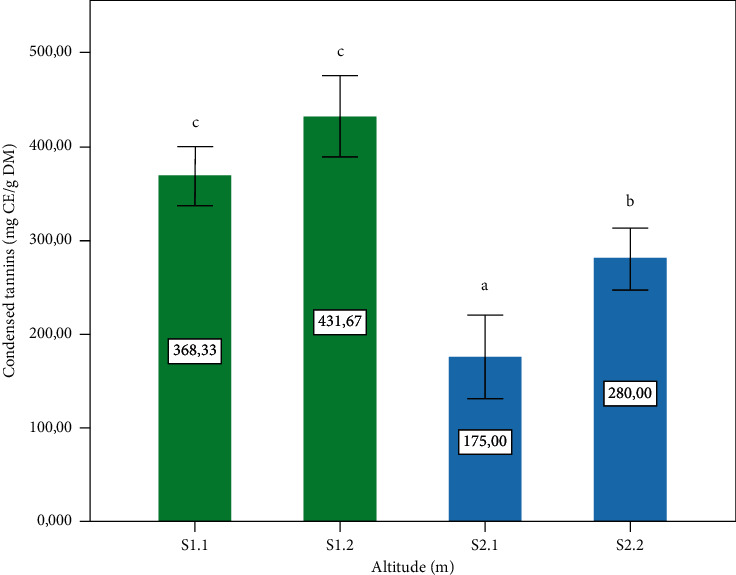
Condensed tannins content of the *P. lentiscus* L. leaves at different altitudes. Data are means of three replicates. Vertical bars represent the standard deviation of the mean. Values not sharing a common letter indicate a significant difference at *p* ≤ 0.05, based on the SNK test. CE: cyanidin equivalent. DM: dry matter. S: site, S1.1 = 1400 m, S1.2 = 1120, S2.1 = 780 m, S2.2 = 720 m.

**Figure 5:**
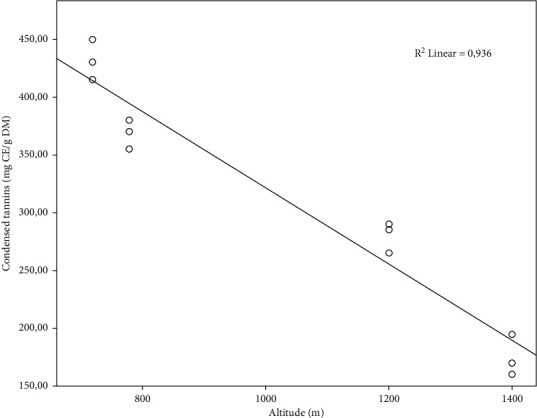
Linear regression curve between condensed tannin and altitudes. CE: cyanidin equivalent. DM: dry matter.

Figure 4 illustrates the concentrations of condensed tannins. The highest concentrations are observed at the level of the S1.1: 1400 m and S1.2: 1120 m, with a significant difference (*p* ≤ 0.05), compared to the concentrations of tannins condensed in the samples from Z2 (Figure 4). Tannins are present in the *P. lentiscus* L. leaves with high contents, especially at high altitudes. In comparison with our result, a study by Atmani et al. [36] reported that the *P. lentiscus* L. leaves showed a high tannins content (909.4 ± 42.61 mg tannic acid equivalents/g dry extract). Several research works have found that the different tissues of these shrubs are rich in tannins, and they have noted a large variation in tannin content [45]. Another study reported beneficial effects of condensed tannins in ruminant feed because they promote the absorption of amino acids in the small intestine by protecting them from the effects of gastric juice, which explains the use of the leaves of this plant to treat a major gastric problem in animals [46]. Furthermore, the condensed tannin concentrations show a significant negative correlation (*r* = −0.967^*∗∗*^) (Table 1) with altitude: when the altitude is high, the tannin content will be less in the leaves (Figure 5).

### 3.5. Ascorbic Acid

**Figure 6:**
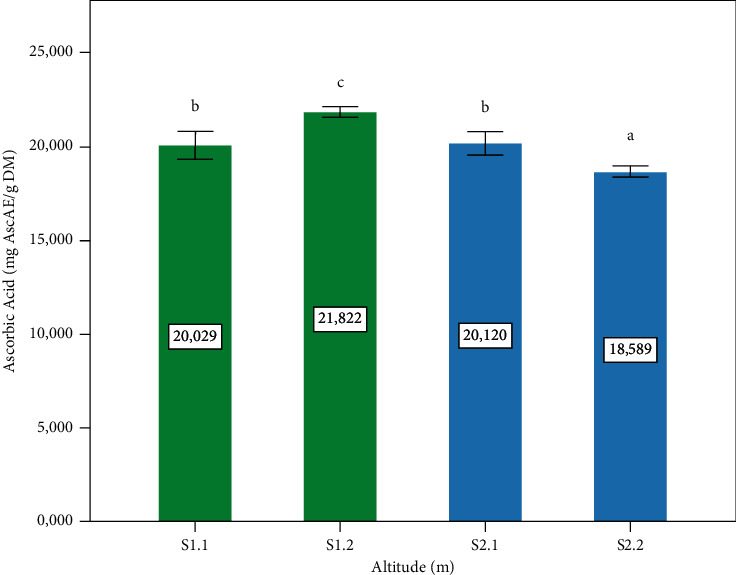
Ascorbic acid content of the *P. lentiscus* L. leaves at different altitudes. Data are means of three replicates. Vertical bars represent the standard deviation of the mean. Values not sharing a common letter indicate a significant difference at *p* ≤ 0.05, based on the SNK test. AscAE: ascorbic acid equivalent. DM: dry matter. S: site, S1.1 = 1400 m, S1.2 = 1120, S2.1 = 780 m, S2.2 = 720 m.

Figure 6 shows the concentrations of ascorbic acid observed at four sites. Site S1.2 gives the maximum value with a significant difference (*p* ≤ 0.05) compared to other sites. The leaves are the primary source of ascorbic acid in plants [47] as a water-soluble antioxidant with exceptional importance in plant cells, protecting plants from oxidative damage [48]. The reducing properties of ascorbic acid come from the enediol group, thanks to which it can directly remove various forms of ROS (reactive oxygen species). However, as a substrate of ascorbate peroxidase, ascorbic acid protects plant cells from oxidative stress and eliminates ROS [49]. Besides, the increase in ascorbic acid synthesis in terms of oxidative stress is associated with its role in the ascorbate-glutathione cycle [50]. However, the concentration of ascorbic acid is high in *P. lentiscus* L. leaves and can be exploited and valorized as a source of vitamin C.

### *3.6. In vitro* Antioxidant Activity

**Figure 7:**
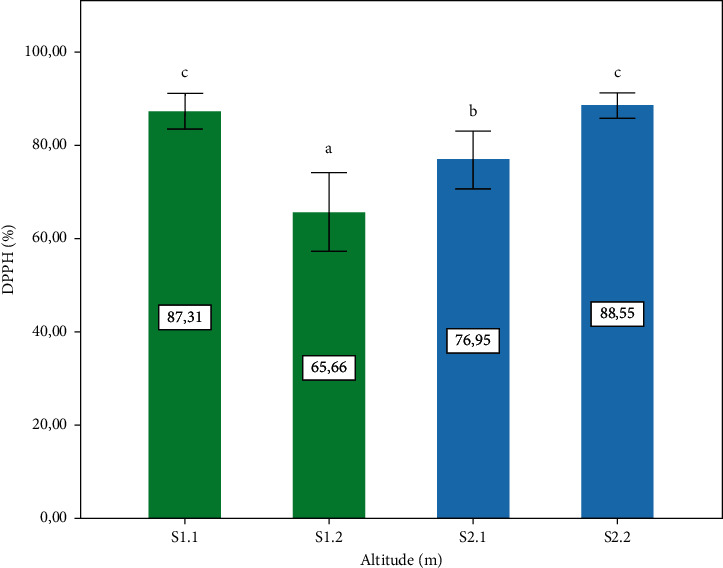
*P. lentiscus* L. leaves' extract on DPPH (%) activity at different altitudes. Data are means of three replicates. Vertical bars represent the standard deviation of the mean. Values not sharing a common letter indicate a significant difference at *p* ≤ 0.05, based on the SNK test. DPPH: 1,1-diphenyl-2-picrylhydrazyl.

**Figure 8:**
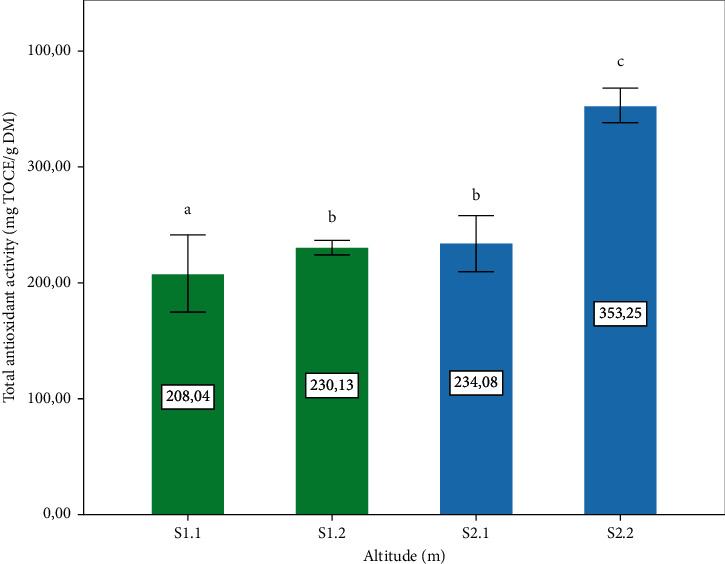
Total antioxidant activity of *P. lentiscus* L. leaves extract expressed in mg equivalent *α*-tocopherol. Vertical bars represent the standard deviation of the mean. Values not sharing a common letter indicate a significant difference at *p* ≤ 0.05, based on the SNK test. TOCE: *α*-tocopherol equivalent. S: site, S1.1 = 1400 m, S1.2 = 1120, S2.1 = 780 m, S2.2 = 720 m S: site, S1.1 = 1400 m, S1.2 = 1120, S2.1 = 780 m, S2.2 = 720 m.

Figure 7 shows the percentage of inhibition activity of DPPH observed in the *P. lentiscus* L. leaves. The highest portions are presented by two altitudes different S1.1: 1400 m and S2.2: 720 m, respectively, with a significant difference (*p* ≤ 0.05), compared to other sites.  Figure 8 shows the total antioxidant activity of the leaves observed in the four-study zone. The highest value was recorded in S2.2 (720 m), with a significant difference (*p* ≤ 0.05) compared to other sites. The antioxidant activity is related to secondary metabolite content analyzed in this study. The analyses of correlation between antioxidant activity and phytochemical content (Table 1) affirmed that the antiradical activity (DPPH) correlated with the content of polyphenols, and this result corresponds with a study confirming that polyphenols are responsible for DPPH activity [51]. However, the total antioxidant activity is correlated with the flavonoid content (Table 1).

## 4. Conclusion

*P. lentiscus* L. leaves' extracts are rich in important chemical compounds, especially phenolic compounds, which confer an important antioxidant power to the plant. The altitude factor had no significant correlation with the contents of the main secondary metabolite other than tannins. Nevertheless, there was a significantly higher content of these metabolites at lower altitudes. However, future research is needed to confirm and enhance our study's high ascorbic acid content.

## Figures and Tables

**Table 1:** Correlation between the analyzed biochemical and antioxidant activity. DPPH: 1,1-diphenyl-2-picrylhydrazyl.

		Polyphenols	Flavonoids	DPPH	Total antioxidant	Tannins	Ascorbic acid
Polyphenols	Pearson correlation	1	0.813^*∗∗*^	0.625^*∗*^	0.534	0.254	−0.566
	Sig. (2-tailed)		0.001	0.030	0.074	0.425	0.055
Flavonoids	Pearson correlation	0.813^*∗∗*^	1	0.415	0.690^*∗*^	0.248	−0.484
	Sig. (2-tailed)	0.001		0.179	0.013	0.438	0.111
DPPH	Pearson correlation	0.625^*∗*^	0.415	1	0.432	−0.310	−0.880^*∗∗*^
	Sig. (2-tailed)	0.030	0.179		0.161	0.326	0.000
Total antioxidant	Pearson correlation	0.534	0.690^*∗*^	0.432	1	−0.262	−0.703^*∗*^
	Sig. (2-tailed)	0.074	0.013	0.161		0.411	0.011
Tannins	Pearson correlation	0.254	0.248	−0.310	−0.262	1	0.555
	Sig. (2-tailed)	0.425	0.438	0.326	0.411		0.061
Ascorbic acid	Pearson correlation	−0.566	−0.484	−0.880^*∗∗*^	−0.703^*∗*^	0.555	1
	Sig. (2-tailed)	0.055	0.111	0.000	0.011	0.061	

^*∗∗*^Correlation is significant at the 0.01 level (2-tailed). ^*∗*^Correlation is significant at the 0.05 level (2-tailed).

## Data Availability

The data used to support the findings of this study are available from the corresponding author upon request.

## References

[1] Mohamed F., Moh R. (2016). Aromatic and medicinal plants of Morocco: richness, diversity and threats. *Bulletin de l’Institut Scientifique, Rabat, Section Sciences de la Vie*.

[2] Kharchoufa L., Bouhrim M., Bencheikh N. (2021). Potential toxicity of medicinal plants inventoried in northeastern Morocco: an ethnobotanical approach. *Plants*.

[3] Ouelbani R., Bensari S., Nardjes T., Khelifi D. (2016). Ethnobotanical investigations on plants used in folk medicine in the regions of Constantine and Mila. *Journal of Ethnopharmacology*.

[4] Ziyyat A., Legssyer A., Mekhfi H., Dassouli A., Serhrouchni M., Benjelloun W. (1997). Phytotherapy of hypertension and diabetes in oriental Morocco. *Journal of Ethnopharmacology*.

[5] Linstadter J., Broich M., Weninger B. (2018). Defining the Early Neolithic of the Eastern Rif, Morocco e Spatial distribution, chronological framework and impact of environmental changes. *Journal: Quaternary International*.

[6] Bozorgi M., Memariani Z., Mobli M., Salehi Surmaghi M. H., Shams-Ardekani M. R., Rahimi R. (2013). Five Pistacia species (P. vera, P. atlantica, P. terebinthus, P. khinjuk, and P. lentiscus): a review of their traditional uses, phytochemistry, and pharmacology. *The Scientific World Journal*.

[7] Dimas K. S., Pantazis P., Ramanujam R. (2012). Chios mastic gum: a plant-produced resin exhibiting numerous diverse pharmaceutical and biomedical pr*ο*perties. *In Vivo*.

[8] Longo L., Scardino A., Vasapollo G. (2007). Identification and quantification of anthocyanins in the berries of *Pistacia lentiscus* L., *Phillyrea latifolia* L. and *Rubia peregrina* L. *Innovative Food Science & Emerging Technologies*.

[9] Boelens M. H., Jimenez R. (1991). Chemical composition of the essential oils from the gum and from various parts ofPistacia lentiscus l. (mastic gum tree). *Flavour and Fragrance Journal*.

[10] Quartu M., Serra M. P., Boi M. (2012). Effect of acute administration of *Pistacia lentiscus* L. essential oil on rat cerebral cortex following transient bilateral common carotid artery occlusion. *Health and Disease*.

[11] Piccolella S., Nocera P., Carillo P. (2016). An apolar Pistacia lentiscus L. leaf extract: GC-MS metabolic profiling and evaluation of cytotoxicity and apoptosis inducing effects on SH-SY5Y and SK-N-BE(2)C cell lines. *Food and Chemical Toxicology*.

[12] Triantafyllou A., Bikineyeva A., Dikalova A., Nazarewicz R., Lerakis S., Dikalov S. (2011). Anti-inflammatory activity of Chios mastic gum is associated with inhibition of TNF-alpha induced oxidative stress. *Nutrition Journal*.

[13] Loutrari H., Magkouta S., Pyriochou A., Koika V., Fragiskos N. (2014). Kolisis, andreas papapetropoulos & charis roussos “mastic oil from Pistacia lentiscus var . chia inhibits growth and survival of human K562 leukemia cells and attenuates angiogenesis. *Nutrition and Cancer*.

[14] Nahida A. N., Ansari S., Siddiqui S. H. (2012). Pistacia lentiscus: a review on phytochemistry and pharmacological properties. *International Journal of Pharmacy and Pharmaceutical Sciences*.

[15] Ezz Eldin H. M., Badawy A. F., Fathy A. (2013). In vitro anti-Trichomonas vaginalis activity of Pistacia lentiscus mastic and Ocimum basilicum essential oil. *Journal of Parasitic Diseases*.

[16] Miyamoto T., Okimoto T., Kuwano M. (2014). Chemical composition of the essential oil of mastic gum and their antibacterial activity against drug-resistant Helicobacter pylori. *Natural Products and Bioprospecting*.

[17] Bampouli A. (2014). Comparison of different extraction methods of Pistacia lentiscus var. chia leaves: yield, antioxidant activity and essential oil chemical composition. *Journal of Applied Research on Medicinal and Aromatic Plants*.

[18] Lichtenthaler H. K., Packer L., Douce R. (1987). Chlorophyll and carotenoids: pigments of photosynthetic biomembranes. *Methods in Enzymol*.

[19] Velioglu Y. S., Mazza G., Gao L., Oomah B. D. (1998). Antioxidant activity and total phenolics in selected fruits, vegetables, and grain products. *Journal of Agricultural and Food Chemistry*.

[20] Koolen H. H. F., Gozzo F. C., de Souza A. Q. L., De Souza A. D. L. (2013). Antioxidant, antimicrobial activities and characterization of phenolic compounds from buriti (Mauritia flexuosa L. f.) by UPLC-ESI-MS/MS. *Food Research International*.

[21] Julkunen-Tiitto R. (1985). Phenolic constituents in the leaves of northern willows: methods for the analysis of certain phenolics. *Journal of Agricultural and Food Chemistry*.

[22] Mau J., Tseng Y., Huang S. (2005). Antioxidant properties of methanolic extracts from *Ganoderma tsugae*. *Food Chemistry*.

[23] De Tommasi N., Pizza C., Di Bari L., Politi M., Morelli I., Braca A. (2002). Antioxidant Principles from Bauhinia tarapotensis. *Journal of Natural Products*.

[24] Umamaheswari T. K. C. M. (2008). In vitro antioxidant activities of the fractions of *Coccinia grandis* l. Leaf extract. *African Journal of Traditional, Complementary and Alternative*.

[25] Chen M., Schliep M., Willows R. D., Cai Z.-L., Neilan B. A., Scheer H. (2010). A red-shifted chlorophyll. *Science*.

[26] Šircelj H., Mikulic-petkovsek M., Veberič R., Hudina M., Slatnar A. (2018). Lipophilic antioxidants in edible weeds from agricultural areas. *Turkish Journal of Agriculture and Forestry*.

[27] Hadif I., Rahim S. A., Sahid I., Rahman A. (2015). Influence of chromium metal on chlorophyll content in leaves of paddy Oryza sativa L. *International Journal of Chemical Sciences*.

[28] Wang C., Zhang Q. (2017). Exogenous salicylic acid alleviates the toxicity of chlorpyrifos in wheat plants (Triticum aestivum). *Ecotoxicology and Environmental Safety*.

[29] Wang J., Lv M., Islam F. (2016). Salicylic acid mediates antioxidant defense system and ABA pathway related gene expression in Oryza sativa against quinclorac toxicity. *Ecotoxicology and Environmental Safety*.

[30] Khoulati A., Ouahhoud S., Bekkouch O., Mamri S., Choukri M. (2021). Impact of the saffron extract on growth and antioxidant enzymes activity of Solanum lycopersicum L. seedlings in Morrocan open field conditions. *Vegetos*.

[31] Khoulati A., Ouahhoud S., Mamri S. (2020). Valorization of Moroccan crocus sativus L . By-products: foliar spraying of aqueous tepal extract stimulates growth and confers antioxidant properties in eggplant seedling under greenhouse conditions. *BioMed Research International*.

[32] Rodríguez-Pérez A. F.-G. C., Quirantes-Piné R., Amessis-Ouchemoukh N., Khodir M., Segura- Carretero A. (2013). A metabolite-profiling approach allows the identification of new compounds from Pistacia lentiscus leaves. *Journal of Pharmaceutical and Biomedical Analysis*.

[33] Boucheffa S., Sobhi W., Attoui A. (2021). Effect of the main constituents of Pistacia lentiscus leaves against the DPPH radical and xanthine oxidase: experimental and theoretical study. *Journal of Biomolecular Structure and Dynamics*.

[34] Chryssavgi G., Vassiliki P., Athanasios M., Kibouris T., Michael K. (2008). Food Chemistry Essential oil composition of *Pistacia lentiscus* L. and *Myrtus communis* L.: evaluation of antioxidant capacity of methanolic extracts. *Food Chemistry*.

[35] Djeridane A., Yousfi M., Nadjemi B., Vidal N., Lesgards J., Stocker P. (2007). Screening of some Algerian medicinal plants for the phenolic compounds and their antioxidant activity. *European Food Research and Technology*.

[36] Atmani D., Chaher N., Berboucha M. (2009). Antioxidant capacity and phenol content of selected Algerian medicinal plants. *Food Chemistry*.

[37] Elez Garofulić I., Kruk V., Martić A. (2020). Evaluation of polyphenolic profile and antioxidant activity of Pistacia lentiscus L. Leaves and fruit extract obtained by optimized microwave-assisted extraction. *Foods*.

[38] Gori A., Nascimento L. B., Ferrini F., Centritto M., Brunetti C. (2020). Seasonal and diurnal variation in leaf phenolics of three medicinal mediterranean wild species: what is the best harvesting moment to obtain the richest and the most antioxidant extracts?. *Molecules*.

[39] Baxter A., Mittler R., Suzuki N. (2013). ROS as key players in plant stress signalling. *Journal of Experimental Botany*.

[40] Kumar S. P. J., Prasad S. R., Banerjee R., Thammineni C. (2015). Seed birth to death: dual functions of reactive oxygen species in seed physiology. *Annals of Botany*.

[41] Soumia K., Tahar D., Lynda L., Saida B., Chabane C., Hafidha M. (2014). Antioxidant A lgeria and antimicrobial activities of selected medicinal plants from. *Journal of Coastal Life Medicine*.

[42] Brunetti C., Di Ferdinando M., Fini A., Pollastri S., Tattini M. (2013). Flavonoids as antioxidants and developmental regulators: relative significance in plants and humans. *International Journal of Molecular Sciences*.

[43] Fraga C. G., Croft K. D., Kennedy D. O., Tomás-Barberán F. A. (2019). The effects of polyphenols and other bioactives on human health. *Food & Function*.

[44] Carretero C. R., Ana M., Lanz D., Matellano L. F., Sánchezb A. R., Castillo L. V. (2001). Phytochemical Analysis of *Phillyrea Latifolia* L. *New Source of Oleuropeoside*.

[45] Ammar K., Zirmi-Zembri N. (2016). Valeur nutritive des principales ressources fourragéres utilisées en Algérie. 2- Les arbres et arbustes fourragers. Livestock Research for Rural Development. *Centro para la Investigación en Sistemas Sostenibles de Producción Agropecuaria*.

[46] Kumar R., Vaithiyanathan S. (1990). Occurrence, nutritional significance and effect on animal productivity of tannins in tree leaves. *Animal Feed Science and Technology*.

[47] Franceschi V. R., Tarlyn N. M. (2015). L-ascorbic acid is accumulated in source leaf phloem and transported to sink tissues in plants. *Plant Physiology*.

[48] Smirnoff N., Sciences B., Laboratories H., Road W. (1996). Botanical briefing: the function and metabolism of ascorbic acid in plants. *Annals of Botany*.

[49] Apel K., Hirt H. (2004). Reactive oxygen species: metabolism, oxidative stress, and signal transduction. *Annual Review of Plant Biology*.

[50] Foyer C. H., Noctor G. (2005). Redox homeostasis and antioxidant signaling: a metabolic interface between stress perception and physiological responses. *The Plant Cell*.

[51] Khoulati A., Ouahhoud S., Mamri S., Alaoui K., Lahmass I. (2020). Annals of Agricultural Sciences Saffron extract stimulates growth , improves the antioxidant components of *Solanum lycopersicum* L., and has an antifungal effect. *Annals of Agricultural Science*.

